# Fibroblast growth factor 21 is associated with widening QRS complex and prolonged corrected QT interval in patients with stable angina

**DOI:** 10.1186/s12872-022-02868-3

**Published:** 2022-09-30

**Authors:** Wei-Chin Hung, Teng-Hung Yu, Chao-Ping Wang, Chia-Chang Hsu, Yung-Chuan Lu, Ching-Ting Wei, Fu-Mei Chung, Yau-Jiunn Lee, Cheng-Ching Wu, Wei-Hua Tang

**Affiliations:** 1 Division of Cardiology, Department of Internal Medicine, E-Da Hospital, No. 1, Yi-Da Rd., Jiau-Shu Village, Yan-Chao Township, Kaohsiung, 82445 Taiwan; 2 School of Medicine, College of Medicine, I-Shou University, Kaohsiung, 82445 Taiwan; 3 School of Medicine for International Students, College of Medicine, I-Shou University, Kaohsiung, 82445 Taiwan; 4 Division of Gastroenterology and Hepatology, Department of Internal Medicine, E-Da Hospital, Kaohsiung, 82445 Taiwan; 5 The School of Chinese Medicine for Post Baccalaureate, College of Medicine, I-Shou University, Kaohsiung, 82445 Taiwan; 6 Division of Endocrinology and Metabolism, Department of Internal Medicine, E-Da Hospital, Kaohsiung, 82445 Taiwan; 7 Division of General Surgery, Department of Surgery, E-Da Hospital, Kaohsiung, 82445 Taiwan; 8 Department of Biomedical Engineering, I-Shou University, Kaohsiung, 82445 Taiwan; 9 Department of Electrical Engineering, I-Shou University, Kaohsiung, 82445 Taiwan; 10 Lee’s Endocrinologic Clinic, Pingtung, 90000 Taiwan; 11 Division of Cardiology, Department of Internal Medicine, Taipei Veterans General Hospital, Yuli Branch, No. 91, Xinxing St., Yuli Township, Hualien County, 981002 Taiwan; 12 Faculty of Medicine, School of Medicine, National Yang Ming Chiao Tung University, Taipei, 112304 Taiwan

**Keywords:** Fibroblast growth factor 21, Wide QRS complex, Prolonged QTc interval, Stable angina

## Abstract

**Background:**

Fibroblast growth factor 21 (FGF21) is produced by cardiac cells, may acts in an autocrine manner, and was suggested to has a cardioprotective role in atherosclerosis. Wide QRS complex and heart rate-corrected QT interval (QTc interval) prolongation are associated to dangerous ventricular arrhythmias and cardiovascular disease mortality. Yet, the role of FGF21 in cardiac arrhythmia has never been studied. The aim of the study was to investigate the relationship between plasma FGF21 and the QRS duration and QTc interval in patients with stable angina.

**Methods:**

Three hundred twenty-one consecutive stable angina patients were investigated. Plasma FGF21 was measured through ELISA, and each subject underwent 12-lead electrocardiography.

**Results:**

FGF21 plasma levels were positively associated with the QRS duration (β = 0.190, *P* = 0.001) and QTc interval (β = 0.277, *P* < 0.0001). With increasing FGF21 tertiles, the patients had higher frequencies of wide QRS complex and prolonged QTc interval. After adjusting for patients’ anthropometric parameters, the corresponding odd ratios (ORs) for wide QRS complex of the medium and high of FGF21 versus the low of FGF21 were 1.39 (95% CI 0.51–3.90) and 4.41 (95% CI 1.84–11.59), respectively, and *p* for trend was 0.001. Furthermore, multiple logistic regression analysis also showed the corresponding odd ratios (ORs) for prolonged QTc interval of the medium and high of FGF21 versus the low of FGF21 were 1.02 (95% CI 0.53–1.78) and 1.93 (95% CI 1.04–3.60) respectively with the *p* for trend of 0.037. In addition, age- and sex-adjusted FGF21 levels were positively associated with fasting glucose, HbA1c, creatinine, and adiponectin, but negatively associated with albumin, and the estimated glomerular filtration rate.

**Conclusions:**

This study indicates that plasma FGF21 is associated with wide QRS complex and prolonged corrected QT interval in stable angina patients, further study is required to investigate the role of plasma FGF21 for the underlying pathogenesis.

**Supplementary Information:**

The online version contains supplementary material available at 10.1186/s12872-022-02868-3.

## Background

The mortality and morbidity of coronary artery disease (CAD) has been decreased after the advance and universal application of either medical and aggressive intravascular intervention therapy of CAD in past decades. Unfortunately, sudden cardiac death still common and difficult to predict in these patients especially in the patient with stable angina who are relative stationary in disease progression [1]. In previous study, the risk factors of sudden death in patients with stable angina mainly focus on the comorbidities of CAD with obesity, peripheral artery disease, left ventricular ejection fraction, low-density lipoprotein cholesterol (LDL-C) levels, alcohol consumption and certain electrocardiographic abnormalities [1, 2]. In 56 years longitudinal, 3983 originally healthy young men prospective study, small electrocardiographic change such as non-specific T wave or ST/T changes were noted to be associated with high short-term risk even sustained excess risk over long term risk for sudden cardiac death [3]. However, the cause of these electrocardiographic abnormalities in patients with stable angina could not be totally explained.

Widening QRS complex greater than 120 ms without bundle branch block (BBB), is related to the slower spread of ventricular depolarization due to reliance on slower muscle-to-muscle spread of depolarization and/or disease of the His-Purkinje network. Recently, wide QRS complex has been proved to have association with an increased risk of malignant ventricular arrhythmia and adverse cardiac events [4]. The QT interval is defined as the duration from the beginning of the Q wave to the end of the T wave. It represents the start of ventricular depolarization to the end of ventricular repolarization and the prolonged corrected QT (QTc) interval is associated with an increased risk of malignant ventricular arrhythmias [5] and sudden death [6]. In addition, recent study showed both of the wide QRS complex and QTc interval prolongation are frequently found in patients with reduced left ventricular ejection fraction and cardiovascular disease with relatively poor prognosis [7, 8].

FGF21 is a previously discovered as a cardiomyokine that regulates lipid and glucose metabolism [9–11]. It is predominantly produced in the liver, and was initially identified as a hepatic endocrine factor that modulates lipid metabolism [12] and promotes thermogenic activity. FGF21 binds to β-Klotho and FGF receptor (FGFR) [13], regulate insulin sensitivity and promote glucose uptake in adipocytes [14, 15]. Recent study showed cardiac FGF21 secretion could be stimulated in response to cardiac stresses although its secretion is lower than the hepatic FGF21 origin [16]. Furthermore, FGF21 could inhibit ischemic arrhythmias by targeting the miR‑143/EGR1 axis [17]. Besides, deletion of FGF21 has been shown in severe myocardial damage [16, 18], and the administration of FGF21 has been proved to prevent ventricular hypertrophy [16] and adverse cardiac remodeling after myocardial infarction (MI) mice model [19]. In contrast, clinical study shown different effects of FGF21. Chou et al. suggested that FGF21 involved in the diastolic heart failure pathophysiology [20] and Shen et al. also reported that among the CAD patients with higher serum level of FGF21, there is a higher incidence of nonfatal myocardial infarction, hospitalization, cardiac death and major adverse cardiovascular events (MACEs) [21, 22]. The effect of FGF21 to cardiovascular diseases is still unclear. In addition, until now, there are few clinical studies investigate the association between FGF21 and cardiac electrical activity in humans. Therefore, here we designed this study to investigate the relationship between FGF21 level and QRS duration and QTc interval in patients with stable angina, in the aim to evaluate the role of FGF21 in these electrocardiographic abnormalities and propose the possible effect of FGF21 to the patient future adverse cardiac events.

## Material and methods

### Study design and participants

There are 321 consecutive consenting patients who underwent angiography for the first time with a clinical diagnosis of stable angina included in this study. The study was taken placed in the cardiovascular department Taiwan E-Da Hospital between January 2007 and December 2016. The inclusion criteria were as follows: (1) stable angina pectoris was defined as effort-related chest tightness without evidence of recent deterioration or rest pain in the previous 6 months as previously described [23, 24]; (2) A successful percutaneous coronary intervention was defined as final angiographic residual stenosis < 30%, without occlusion > 1 mm coronary branch or flow-limiting dissection and with the resulting Thrombolysis in Myocardial Infarction grade 3, by quantitative coronary angiography [25]. Patients with following criteria were excluded in our study: (1) a recent inflammatory diseases (such as sepsis or infection), liver diseases, collagen diseases, malignancy or steroid use; (2) a history of psychosis with and without psychotropics; (3) history of myocardial infarction, heart valve disease, and heart surgery; (4) EKG with BBB pattern, fibrillation or flutter, and atrioventricular blocks; (5) recent use of medications which has prolong QT interval effect, such as class I anti-arrhythmics (e.g. quinidine, mexiletine, etc.), class III anti-arrhythmics (e.g. amiodarone, vernakalant, etc.), certain psychotropic drug, certain microcrobial and antimalarial drugs; (6) electrolytes imbalance.

### Baseline data collection

A detailed interview was conducted with each patient about their demographic characteristics, as well as their medical and personal history before the coronary angiography examination. Three smoking status was classified: never having smoked, former smoker (ceased smoking for at least 1 year), or current smoker. Former and current smokers were analyzed as one group in our study. Patient’s blood pressure was measured in a sitting position, after rested for 5 min, by a trained assistant, with a digital automatic blood pressure monitor (model HEM-907; Omron, Omron, Japan). Body height and body weight were measured to the nearest 0.1 cm and 0.1 kg respectively using an electronic scale with participants wearing a hospital gown. Waist and hip circumferences were measured at the narrowest point between the lowest rib and the uppermost lateral border of the right iliac crest to the nearest 0.1 cm. The hips were measured at the widest point. Body mass index (BMI) was estimated by dividing the body weight in kilograms by the square of the body height in meters by definition.

### Laboratory measurements

Peripheral blood samples were taken after the subject had fasted for 8 h and before the coronary angiography. Complete blood cell counts, general biochemical measurement (including serum creatinine, electrolytes, uric acid, albumin, glucose, serum alanine aminotransferase (ALT) and aspartate aminotransferase (AST)) and lipid profiles(total cholesterol, plasma triglycerides (TGs), low-density lipoprotein cholesterol (LDL-C), and high-density lipoprotein cholesterol (HDL-C)) were measured in all patients using standard commercial methods on a parallel, multi-channel analyzer (Hitachi 7170A, Tokyo, Japan) according to the International Federation of Clinical Chemistry methods. High performance liquid chromatography was used to measure Hemoglobin A1c (HbA1c). The concentration of plasma FGF21 was measured by enzyme-linked immunosorbent assay (ELISA) kits (Cloud-Clone Corp., Katy, USA). The dilution and standard curves were parallel. The intra- and inter-assay coefficients of variation for the assay were < 10% (n = 3) and < 12% (n = 3). In addition, ELISA was also used to measure high sensitivity C-reactive protein (hs-CRP) and adiponectin levels. Full details of how the bloodwork was analysed are included in the Additional file 1 [26].

### Definitions

Type 2 diabetes was defined according to the World Health Organization criteria as a history of type 2 diabetes and/or the subject receiving anti diabetics medical therapy [27]. Hypertension was defined as a resting systolic/diastolic blood pressure (SBP/DBP) of ≥ 140 mmHg/ ≥ 90 mmHg, or recent us of antihypertensive drugs. Hyperlipidemia was defined as elevated triglycerides (≥ 150 mg/dl), and/or low HDL-C (< 35 mg/dl in men or < 39 mg/dl in women), and/or elevated total cholesterol (≥ 200 mg/dl), and/or elevated LDL-C (≥ 130 mg/dl), or recent receiving treatment for a lipid disorder according to NCEP ATP III [28]. Left ventricular mass was calculated using a two-dimensional method and indexed to the body surface area. Left ventricular hypertrophy (LVH) was defined as a left ventricular mass index of > 131 g/m^2^ for men and > 100 g/m^2^ for women [29]. Furthermore, estimated glomerular filtration rate (eGFR) was calculated using the Chronic Kidney Disease Epidemiology Collaboration (CKD-EPI) equation within 3–6 months of admission [30]. In addition, we recorded the occurrence of MACEs after the patients were discharged from the hospital. MACEs were defined as all-cause mortality, cardiovascular-related re-hospitalization (including heart failure, non-fatal reinfarction, uncontrolled angina) or repeated percutaneous coronary interventions/coronary artery bypass grafting.

### Angiographic definitions

Before performing coronary angiography, noninvasive stressing test such as treadmill exercise test or myocardial perfusion scintigraphy was performed to the patients at our cardiovascular outpatient department to evaluate the possibility of CAD and indication of coronary angiography. If there was a positive result, further coronary angiography was suggested and underwent if patient agreed. Coronary angiograms were performed according to standard techniques. The coronary artery stenosis severity was assessed using quantitative angiography. Quantitative coronary angiographic and angiograms analyses were evaluated by at least two experienced interventional cardiologists who were blinded to the patient’s clinical history and information. Number of diseased coronary vessels and Gensini Score were determined and full information on the scoring systems are detailed in the Additional file 1 [31].

### Electrocardiography, QT and QTc interval measurements

The QT interval and QRS duration were manually measured by 2 cardiologists who were blinded to the study aims. A twelve-lead ECG was performed using standardized protocol at baseline at supine position after 15 min rest during the patient admission physical examination. Standard intervals (heart rate, PR, QRS, and QT) and amplitudes (R, S, and T waves) were analyzed using standard protocols as detailed in the Additional file 1 [32–34]. The QT interval was measured from the beginning of the QRS complex to the end of the T wave in the electrocardiogram. Details of the QTc measurement please refer to the Additional file 1 [35, 36]. Prolonged QTc was defined as in men ≥ 451 ms or women ≥ 471 ms [37]. Abnormal wide QRS complex was defined as a QRS duration > 120 ms, but exclude those typical BBB pattern (right BBB defined as QRS duration ≥ 120 ms with large R’-wave in V1/V2 with a broad deep S-wave in V5/V6, left BBB defined as QRS duration ≥ 120 ms with deep broad S-wave in V1/V2 and broad clumsy R-wave in V5/V6).

### Statistical analysis

Normally distributed continuous variables are presented in mean ± standard deviation (SD), and non-normally distributed continuous variables are presented in median (interquartile range (IQR)). Data normality was assessed by Kolmogorov- Smirnov test. Before statistical tests were performed, the serum levels of TGs, AST, ALT, creatinine, hs-CRP, adiponectin, and FGF21 were logarithmically transformed to achieve a normal distribution. One-way ANOVA for variables of normal distribution followed by the Tukey pairwise comparison was used to compare the differences in continuous variables between the FGF21 tertiles. Categorical variables are presented in frequencies with percentages and the inter-group comparisons were tested using chi-square test.

We further divided the distribution of FGF21 into tertile and estimate the odds ratios (ORs) of wide QRS complex and prolonged QTc interval using logistic regression models. In the analysis, we use the lowest tertile as the reference category. Multivariate adjusted ORs are presented with 95% confidence interval (CI). The correlations and independence between plasma FGF21 levels and the values of other parameters were examined by Pearson’s correlation coefficient and multiple linear regression analyses. All tests were two-tailed. A *p*-value < 0.05 was considered to be statistically significant. All data were analyzed using JMP version 7.0 for Windows (SAS Institute, Cary, NC, USA).

## Results

### Main characteristics

**Fig. 1:**
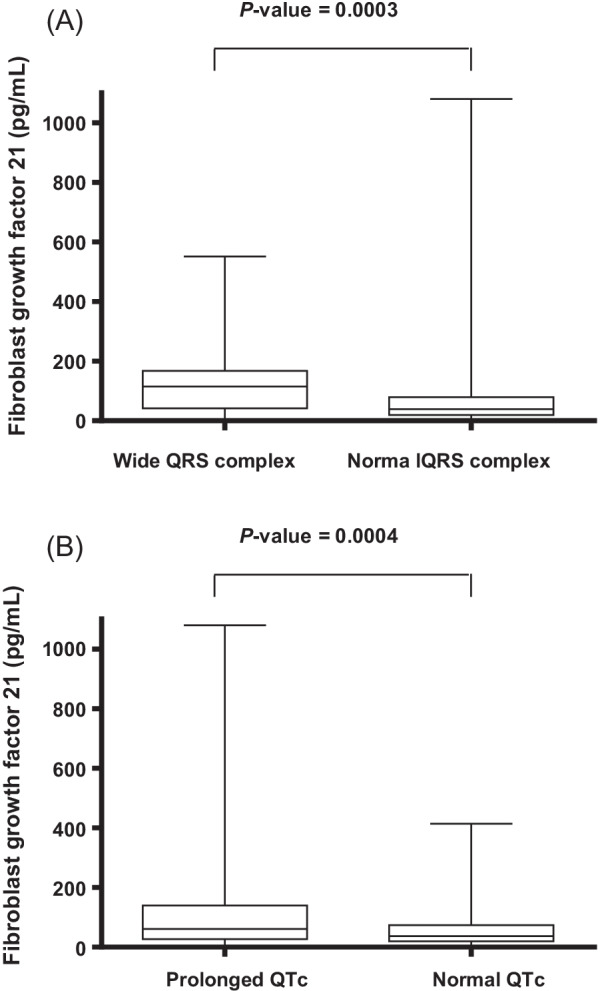
Patients with wide QRS complex had significantly higher fibroblast growth factor 21 (FGF21) compared to patients with normal QRS complex (**A**). Patients with prolonged QTc interval had significantly higher FGF21 compared to patients with normal QTc interval (**B**). The bottom of the box plots indicates the 25th percentile and the top indicates the 75th percentile. The middle line of the box indicates the median. The lower and upper ends of the whiskers indicate the minimum and maximum observations below the upper fence (1.5 interquartile range above the 75th percentile), respectively

There are 321 patients enrolled in this study. 75% (241) of the participants were male with a mean age of 69.0 years (SD, 12.1 years). The mean QTc interval and QRS duration of all participants were 447 ms and 98 ms (SD, 41 ms and 21 ms). The plasma FGF21 levels were significantly higher in the patients with wide QRS complex than in those normal QRS complex [114.9 pg/mL (interquartile range, 41.8 pg/mL to 167.5 pg/mL) vs. 38.8 pg/mL (interquartile range, 19.4 pg/mL to 79.5 pg/mL), Fig. 1A]. Furthermore, the plasma levels of FGF21 were significantly higher in the patients with prolonged QTc interval [61.0 pg/mL (interquartile range, 27.5 pg/mL to 140.1 pg/mL) with prolonged QTc vs. 37.4 pg/mL (interquartile range, 20.1 pg/mL to 73.5 pg/mL) with normal QTc, Fig. 1B]. Table 1 shows the clinical characteristics of studied participants stratified by FGF21. The mean FGF21 level was 85.2 pg/mL, and the median plasma FGF21 level was 42.4 pg/mL (interquartile range, 21.1 pg/mL to 100.2 pg/mL). All the subjects were divided according to tertiles of FGF21 as follows: low FGF21 (≤ 28.5 pg/mL), n = 108; medium FGF21 (28.6 pg/mL to 65.8 pg/mL), n = 106; and high FGF21 (> 65.8 pg/mL), n = 107. The high FGF21 patients were older and had higher rates of LVH, all-cause mortality, wide QRS complex, and prolonged QTc. Furthermore, these patients also had higher levels of waist-to-hip ratio, heart rate, and QRS duration than those with the low FGF21 group. Moreover, Longer QTc interval was also noted in the high FGF21 group than the medium and low FGF21 groups. In addition, the patients in the high FGF21 group also had lower rates of hyperlipidemia, current smoker, and statin medication, as well as lower left ventricular ejection fraction (LVEF) compared with those who had the low FGF21 group.

**Table 1:** Main characteristics according to tertiles of fibroblast growth factor 21

Parameter	Low FGF21 ≤ 28.5 pg/mL	Medium FGF21 = 28.6–65.8 pg/mL	High FGF21 > 65.8 pg/mL	*P*-value
Number	108	106	107	
Sex (male/female)	82/26	84/22	75/32	0.294
Age (years)	66.5 ± 12.0	68.0 ± 11.8	72.5 ± 11.6	0.001
Hypertension (n, %)	76(70.4)	78(73.6)	80(74.8)	0.754
Hyperlipidemia (n, %)	71(65.7)	75(70.8)	56(52.3)	0.016
Diabetes mellitus (n, %)	37(34.3)	50(47.2)	51(47.7)	0.079
Left ventricular hypertrophy (n, %)	50(46.3)	52(49.1)	72(67.3)	0.032
MACEs (n, %)	45(41.7)	59(55.7)	49(45.8)	0.109
All-cause Mortality (n, %)	8(7.4)	14(13.2)	21(19.6)	0.031
Current smoker (n, %)	46(42.6)	50(47.2)	33(30.8)	0.043
Body mass index (kg/m^2^)	25.7 ± 3.9	26.1 ± 3.6	24.7 ± 4.5	0.044
Waist-to-hip ratio	0.93 ± 0.07	0.94 ± 0.06	0.97 ± 0.12	0.010
Systolic BP (mmHg)	130 ± 22	131 ± 22	130 ± 26	0.926
Diastolic BP (mmHg)	76 ± 14	77 ± 12	77 ± 15	0.992
LVEF (%)	60.8 ± 15.1	58.8 ± 15.5	55.5 ± 16.0	0.045
Electrocardiography parameters				
Heart rate (bpm)	74.0 ± 17.6	77.6 ± 20.0	83.1 ± 20.8	0.003
PR interval (ms)	167.5 ± 30.4	169.4 ± 26.5	172.7 ± 39.7	0.540
QRS duration (ms)	93.7 ± 15.8	98.4 ± 22.1	103.1 ± 22.2	0.004
QT interval (ms)	394.7 ± 46.0	399.3 ± 44.9	401.1 ± 57.9	0.626
QTc interval (ms)	434.2 ± 35.6	444.5 ± 35.6	461.9 ± 45.3	< 0.0001
Wide QRS complex^†^ (n, %)	8(7.4)	12(11.3)	33(30.8)	< 0.0001
Prolonged QTc interval^‡^ (n, %)	30(27.8)	32(30.2)	51(47.7)	0.004
Medication (n, %)				
Anti-arrhythmic medication	10(9.3)	8(7.6)	10(9.4)	0.871
Beta-blockers	58(53.7)	57(53.8)	49(45.8)	0.406
Diuretics	31(28.7)	33(31.1)	31(29.0)	0.913
Statins	66(61.1)	59(55.7)	45(42.1)	0.016
Number of diseased vessels (%)				
Single-vessel disease	35(32.4)	33(31.1)	28(26.2)	0.574
Two-vessel disease	24(22.2)	36(34.0)	39(36.5)	0.054
Three-vessel disease	49(45.4)	37(34.9)	40(37.4)	0.260

Data are expressed as the mean ± SD, number (percentage), or median (interquartile range). *FGF* fibroblast growth factor; *QTc* corrected QT; *LVEF* left ventricular ejection fraction; *MACEs* major adverse cardiovascular events

^†^Wide QRS complex was defined as a QRS duration > 120 ms

^‡^Prolonged QTc was defined as a men ≥ 451 ms or women ≥ 471 ms

### Biochemical characteristics

In Table 2, with the increase of FGF21 tertile, there were significant decreases of eGFR, hemoglobin, and albumin concentrations. In the other hand, the increasing of FGF21 tertile also found in significant increases of fasting glucose, HbA1c, blood urine nitrogen (BUN), creatinine, hs-CRP, and adiponectin concentrations.

**Table 2:** Biochemical characteristics according to tertiles of fibroblast growth factor 21

Parameter	Low FGF21 ≤ 28.5 pg/mL	Medium FGF21 = 28.6–65.8 pg/mL	High FGF21 > 65.8 pg/mL	*P*-value
Number	108	106	107	
Total-cholesterol (mg/dl)	171.0 ± 44.5	177.1 ± 45.6	171.3 ± 46.1	0.548
Triglycerides (mg/dl)	96.5(70.3–154.8)	120.0(75.5–174.0)	127.0(87.0–183.0)	0.104
HDL-cholesterol (mg/dl)	40.4 ± 10.5	38.4 ± 13.2	39.0 ± 12.0	0.467
LDL-cholesterol (mg/dl)	103.6 ± 36.5	108.5 ± 38.9	97.6 ± 38.4	0.116
AST (U/L)	32.0(25.0–46.5)	32.0(23.0–43.3)	35.0(25.0–70.0)	0.060
ALT (U/L)	30.0(19.0–43.8)	31.0(19.0–46.8)	27.0(17.0–52.0)	0.281
Fasting glucose (mg/dl)	138.3 ± 67.6	151.3 ± 81.3	170.7 ± 94.6	0.016
HbA1c (%)	6.6 ± 1.5	7.1 ± 1.8	7.3 ± 1.8	0.027
Uric acid (mg/dl)	7.3 ± 6.4	7.2 ± 2.4	7.2 ± 2.5	0.988
BUN (mg/dl)	19.9 ± 7.1	23.8 ± 16.5	32.0 ± 20.9	< 0.0001
Creatinine (mg/dl)	1.1(1.1–1.4)	1.3(1.1–1.6)	1.7(1.3–3.6)	< 0.0001
Estimated GFR (ml/min/1.73m^2^)	65.3 ± 18.1	56.6 ± 22.0	39.2 ± 24.3	< 0.0001
Sodium (meq/l)	138.9 ± 3.6	138.8 ± 4.3	138.3 ± 4.3	0.480
Potassium (meq/l)	3.8 ± 0.4	3.8 ± 0.8	4.0 ± 0.7	0.158
Calcium (mg/dl)	8.7 ± 0.5	8.7 ± 1.0	8.8 ± 1.0	0.576
Hemoglobin (g/dl)	13.6 ± 1.9	13.4 ± 2.2	12.2 ± 2.4	< 0.0001
Albumin (g/dl)	4.0 ± 0.6	3.8 ± 0.5	3.7 ± 0.5	0.0004
Hs-CRP (mg/l)	1.5(0.7–5.1)	3.1(1.2–10.0)	4.3(1.5–15.0)	0.044
Adiponectin (μg/ml)	4.0(1.8–10.7)	4.2(1.6–6.5)	6.9(3.7–13.4)	0.0002

Data are expressed as the mean ± SD, number (percentage), or median (interquartile range). *FGF* fibroblast growth factor; *HDL* high-density lipoprotein; *LDL* low-density lipoprotein; *AST* aspartate aminotransferase; *ALT* alanine aminotransferase; *BUN* blood urea nitrogen; *GFR* glomerular filtration rate; *Hs-CRP* high-sensitivity C-reactive protein

### Association of the FGF21 for the Wide QRS Complex and Prolonged QTc Interval

Table 3 shows results the ORs of the wide QRS complex and prolonged QTc interval estimation according to the FGF21 tertiles. Compared with subjects with low of FGF21, the ORs of wide QRS complex for medium and high of FGF21 were 1.60 (95%CI 0.63–4.24) and 5.57 (95%CI 2.54–13.61) respectively. Using multiple logistic regression with adjustment, the corresponding ORs of the wide QRS complex for medium and high of FGF21 versus low of FGF21 still high ((1.39 (95%CI 0.51–3.90) and 4.41 (95%CI 1.84–11.59) respectively). Similarly, compared to the participants with low of FGF21, the ORs of prolonged QTc interval for medium and high of FGF21 were 1.12 (95%CI 0.62–2.04) and 2.37 (95%CI 1.35–4.21). After adjustment in the multiple logistic regression, the corresponding ORs of the prolonged QTc interval for medium and high of FGF21 versus low of FGF21 remained 1.02 (95%CI 0.53–1.78) and 1.93 (95%CI 1.04–3.60).

**Table 3:** ORs for wide QRS complex and prolonged QTc interval according to fibroblast growth factor 21 tertiles (95% CI)

Factor	Low FGF21 ≤ 28.5 pg/mL	Medium FGF21 = 28.6–65.8 pg/mL	High FGF21 > 65.8 pg/mL	*P* for trend
Number	108	106	107	
Wide QRS complex				
Model 1	1.00	1.60 (0.63–4.24)	5.57 (2.54–13.61)	< 0.0001
Model 2	1.00	1.54 (0.61–4.10)	5.16 (2.32–12.74)	< 0.0001
Model 3	1.00	1.54 (0.59–4.19)	4.92 (2.18–12.27)	< 0.0001
Model 4	1.00	1.39 (0.51–3.90)	4.41 (1.84–11.59)	0.001
Prolonged QTc interval				
Model 1	1.00	1.12 (0.62–2.04)	2.37 (1.35–4.21)	0.003
Model 2	1.00	1.09 (0.60–1.98)	2.08 (1.17–3.74)	0.013
Model 3	1.00	1.05 (0.57–1.95)	2.14 (1.19–3.89)	0.011
Model 4	1.00	1.02 (0.53–1.78)	1.93 (1.04–3.60)	0.037

Model 1 unadjusted model, Model 2 adjusted for age and sex, Model 3 additionally adjusted for body mass index, total-cholesterol, potassium, and smoking, Model 4 additionally adjusted for diabetes mellitus, hypertension, chronic kidney disease, statins medication. *FGF* fibroblast growth factor

### Association between plasma FGF21 levels and clinical laboratory data

Pearson’s correlation analysis showed the plasma FGF21 levels were positively correlated with age, heart rate, QRS duration, QTc interval, fasting glucose, HbA1c, BUN, creatinine, hs-CRP, and adiponectin. However, they were negatively correlated with LVEF, BMI, current smoking, LDL-C, albumin, and eGFR (Table 4). Furthermore, age- and sex-adjusted FGF21 levels were significantly positively associated with heart rate, QRS duration, QTc interval, fasting glucose, HbA1c, BUN, creatinine, and adiponectin. In contrast, they were significantly negatively associated with current smoking, LDL-C, albumin, and eGFR. Moreover, age and sex-adjusted FGF21 levels were not significantly correlated with PR interval, QT interval, LVEF, left ventricular mass index, SBP, DBP, BMI, waist circumference, total cholesterol, TGs, HDL-C, and hs-CRP in our analysis (Table 4).

**Table 4:** Association between plasma fibroblast growth factor 21 levels and clinical laboratory data

Characteristic	Model 1		Model 2	
	r	*P*-value	β	*P*-value
Age	0.174	0.002	–	–
Male sex	− 0.069	0.217	–	–
Heart rate	0.184	0.001	0.155	0.006
PR interval	0.086	0.150	0.074	0.209
QRS duration	0.191	0.001	0.180	0.001
QT interval	0.038	0.500	0.024	0.667
Corrected QT interval	0.277	< 0.0001	0.247	< 0.0001
LVEF	− 0.127	0.035	− 0.094	0.125
Left ventricular mass index	0.126	0.089	0.130	0.083
Systolic blood pressure	− 0.023	0.681	− 0.041	0.472
Diastolic blood pressure	− 0.012	0.839	− 0.009	0.873
Body mass index	− 0.125	0.026	− 0.092	0.115
Waist circumference	− 0.040	0.551	− 0.045	0.513
Current smoking	− 0.176	0.002	− 0.167	0.007
Total-cholesterol	− 0.070	0.217	− 0.048	0.398
Triglycerides	0.058	0.299	0.112	0.052
HDL-cholesterol	− 0.041	0.462	− 0.050	0.368
LDL-cholesterol	− 0.168	0.003	− 0.155	0.006
Fasting glucose	0.180	0.001	0.170	0.002
HbA1c	0.149	0.016	0.152	0.014
Albumin	− 0.239	< 0.0001	− 0.204	0.001
Blood urea nitrogen	0.344	< 0.0001	0.331	< 0.0001
Creatinine	0.526	< 0.0001	0.522	< 0.0001
Estimated GFR	− 0.483	< 0.0001	− 0.566	< 0.0001
Hs-CRP	0.130	0.023	0.103	0.074
Adiponectin	0.256	< 0.0001	0.222	< 0.0001

Model 1: Pearson correlation coefficient. Model 2: Regression coefficient adjusted for age and sex. *LVEF* left ventricular ejection fraction; *HDL* high-density lipoprotein; *LDL* low-density lipoprotein; *GFR* glomerular filtration rate; *Hs-CRP* high-sensitivity C-reactive protein

### Type 2 diabetes mellitus (T2DM) affects plasma levels of FGF21

**Fig. 2:**
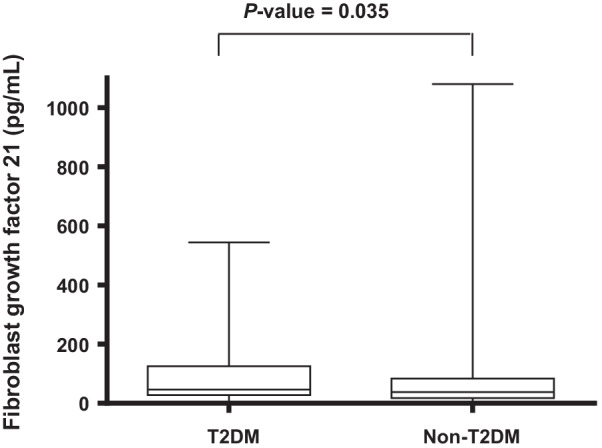
Patients with type 2 diabetes mellitus (T2DM) had significantly higher fibroblast growth factor 21 levels compared to patients without T2DM. The bottom of the box plots indicates the 25th percentile and the top indicates the 75th percentile. The middle line of the box indicates the median. The lower and upper ends of the whiskers indicate the minimum and maximum observations below the upper fence (1.5 interquartile range above the 75th percentile), respectively

**Fig. 3:**
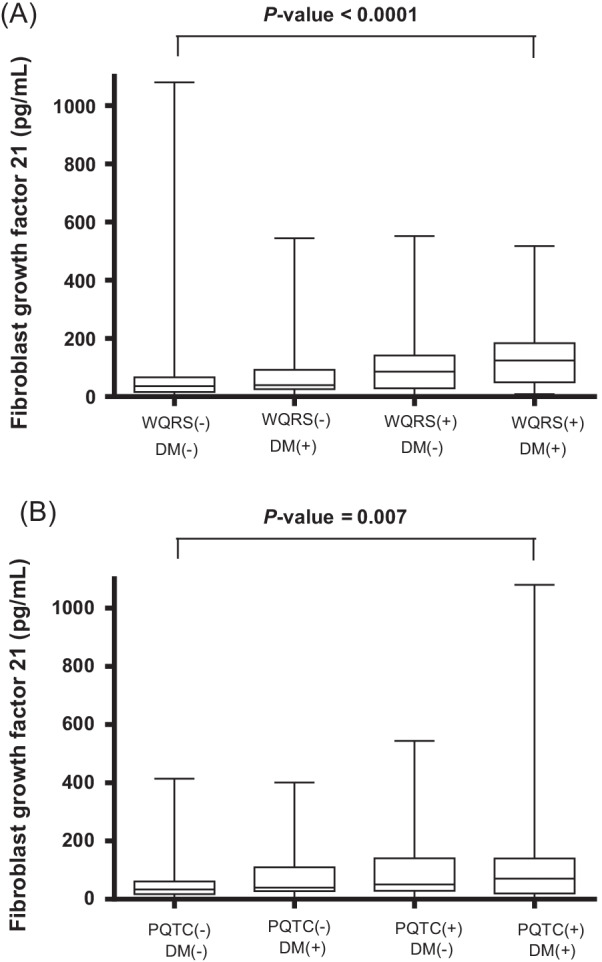
The patients with a wide QRS complex and type 2 diabetes mellitus (T2DM) had higher levels of fibroblast growth factor 21 (FGF21) than those without a wide QRS complex and T2DM and those without a wide QRS complex and without T2DM (**A**). Furthermore, the patients with a prolonged QTc interval and T2DM had higher levels of FGF21 than those without a prolonged QTc interval with T2DM and those without a prolonged QTc interval and without T2DM (**B**). Differences between groups were analyzed by one-way analysis of variance, followed by Tukey’s pairwise comparison. The bottom of the box plots indicates the 25th percentile and the top indicates the 75th percentile. The middle line of the box indicates the median. The lower and upper ends of the whiskers indicate the minimum and maximum observations below the upper fence (1.5 interquartile range above the 75th percentile), respectively

In the patients with T2DM, the levels of plasma FGF21 were significantly higher than in those without T2DM [46.6 pg/mL (interquartile range, 28.2 pg/mL to 125.1 pg/mL) vs. 38.1 pg/mL (interquartile range, 17.6 pg/mL to 84.1 pg/mL), p = 0.035; Fig. 2]. Furthermore, the patients with a wide QRS complex and T2DM had higher levels of FGF21 than those with normal QRS complex with and without T2DM, but not those with wide QRS complex without T2DM (Fig. 3A). Moreover, the T2DM patients with a prolonged QTc interval had higher levels of FGF21 than those with normal QTc interval with or without T2DM, but not those with prolonged QTc interval without T2DM (Fig. 3B).

## Discussion

Among 321 stable angina patients in our study, there are 16% of the patients has wide QRS without BBB and near one third of them have a prolonged QTc by definition. We found the plasma FGF21 level was increased in patients with a wide QRS complex and prolonged QTc interval with and without adjusted model. From our analysis, the plasma FGF21 level was positively associated with fasting glucose, HbA1c, creatinine and adiponectin, but negatively associated with albumin and eGFR. To the best of our knowledge, this is the first study to show an association between increased plasma FGF21 level and wide QRS complex and prolonged QTc interval in human subjects.

The biological effect of FGF21 on CAD are not well understood. Literatures review showed a higher FGF21 in patient with diastolic heart failure or CAD with greater risk of MACEs [21, 22] but have a cardio protecting and antiarrhythmic effect in animal studies [16–19]. This conflict observation is interesting. In previous animal study, FGF21 treatment reduced susceptibility to arrhythmia in infarcted mice hearts by suppressing miR-143 expression and regulate the EGR1-SCN5A /KCNJ2 pathway in MI [17]. However, some vivo experiments, FGF21 levels was found to be correlated with the dysregulated metabolic status. For examples, FGF21 resistance has been observed in in vivo experiments with DIO mice livers and white adipose tissue [38], and another report in ex vivo experiments with obese rat hearts [39]. Furthermore, FGF21 resistance has also been observed in clinical reports where the serum FGF21 level was significantly increased in patients with nonalcoholic fatty liver disease (NAFLD) [40], coronary heart disease [40, 41], metabolic syndrome [42], and T2DM [43]. While FGF21 mainly secreted by the liver which regulates insulin sensitivity and glucose homeostasis [44]. FGF21 has also been identified in the heart as a paracrine signal protein [45]. In one clinical study, FGF21 is found abundantly secreted into the plasma in response to cardiac stress stimuli in patients with cardiovascular diseases [46].

QRS complex is modified by various mechanisms and clinical conditions. A wide QRS complex may be related to obesity [47], cigarette smoking [48], hypertension [47], NAFLD [49], diabetes mellitus [50], metabolic syndrome [51], heart failure [52], CKD [53], and inflammation [54]. As patient with heart diseases always has metabolic dysregulation problems, high serum FGF21 levels in these patients are reasonable. Therefore, we suggest serum FGF21 levels could be considered an indicator of adverse metabolic dysregulation but not the explanation of the cause of these abnormal cardiac function parameters findings.

Previous studies reported there is an increase of FGF21 levels in cardio-metabolic conditions such as CAD, heart failure, atrial fibrillation, MI, obesity, and diabetes mellitus [55, 56]. In recent one study regarding the influence of lifestyle to the FGF21 serum level. It showed the age, aminotransferase, gamma-glutamyl transpeptidase, smoking status, and breakfast and alcohol consumption frequency were independently associated with FGF21 levels. More interestingly, the FGF21 levels were found more profound correlated with waist circumference, SBP, and total cholesterol in the relative healthy the non-obese group [57]. These findings are consistent with our study result. With increasing FGF21 tertiles, we found our patients had a higher waist-to-hip ratio and HbA1c as well as higher prevalence of LVH and incrementally lower LVEF.

In our study, we found that the QT interval is prolonged with the increase of FGF21 levels. QT interval prolongation has been associated with diabetes, obesity, and adiposity, cardiovascular diseases which are all related to an increased FGF21 level [40]. Yılmaz et al. further suggested regional adipose tissue deposition especially epicardial fat volume may play an important role in QT interval prolongation pathogenesis [58]. As FGF21 was identified as a hepatic endocrine factor which modulates lipid metabolism, and the serum FGF21 level progressively increased with visceral fat, and associated with NAFLD [59], it is reasonable to proposed there is a possible link between FGF21 and prolonged QTc interval via the lipid metabolism happened at epicardial fatty tissue. However, like the phenomenon we found in QRS interval. FGF21 might not directly induced the prolonged QTc interval but reflect the complex underlying metabolic dysregulation and comorbidity of the patients.

In our study, we found that a high FGF21 level was significantly associated with all-cause mortality in stable angina patients which similar to previous observations. FGF21 levels were known significantly correlated with left ventricular systolic dysfunction at baseline and has a greater risk to develop MACEs and cardiac death [21, 60]. Furthermore, elevated circulating FGF21 levels also found in patients with carotid atherosclerosis [61], subclinical atherosclerosis [62], CHD [41], and acute MI [63]. All these data logically conduct the idea that high levels of serum FGF21 may be indicative for the adverse cardiovascular events following CAD. However, because of the relative short duration of follow-up and small sample size, further studies with a longer follow-up period and larger patient population are still required to confirm this finding.

Interestingly, we found that T2DM patients had significantly higher FGF21 levels compared to patients without T2DM in our study. The T2DM patients with a wide QRS complex had higher levels of FGF21 than those with normal QRS complex with and without T2DM. Moreover, the patients with a prolonged QTc interval and T2DM also had higher levels of FGF21 than those with normal QTc interval with or without T2DM. Recently, few studies have shown the early compensatory high serum level of FGF21 levels might be responsive to the occurrence and development of DM-induced cardiovascular complications [62, 64]. In addition, the deletion of FGF21 has also been proved to have the relationship with the aggravation of DM-induced cardiovascular injury in some reports [18, 65].

In our study, a higher FGF21 level was positively associated with plasma adiponectin level. Adiponectin and FGF21 both control the metabolism of lipids and carbohydrates, which is essential for the maintenance of energy homeostasis in the body and hence for survival. In one of our previous studies, plasma adiponectin levels have been proved to have the correlation with QTc interval prolongation [67]. A previous study further demonstrated adiponectin could mediates the metabolic effects of FGF21 [66]. In addition, insulin resistance, which is associated with adiponectin resistance, also found could predict a future increase in Tpeak-Tend interval which will influence QT interval in the general population [68].

Therefore, on the basis with all our findings and other reports, we hypotheses the mechanisms that may be involved the effects of an elevated FGF21 level to the electrocardiographic abnormalities we observed. First, the increase of serum FGF21 may be associated with the corresponding metabolic dysregulation. Second, in individuals with metabolic abnormalities, the FGF21 signaling pathway may be impaired, leading to FGF21 resistance. As the more severe of the underlying metabolic abnormalities, the secretion of FGF21 increased and the associated electrocardiographic abnormalities become obvious.

There were some limitations to the current study. First, the cross-sectional design of our study limits the ability to infer any causal relationships between FGF21 level and wide QRS complex and QTc prolongation and limited the robust comparisons. Further case controlled and cohort studies are required. Second, we did not determine the 24-h Holter and more complex cardiac electroactivity parameters such as heart rate variability, PR and QT interval variability in this report, further calculation should be performed in the future to evaluate relevant arrhythmias and the sympathetic and parasympathetic effect of the FGF21. Third, although the ST-T manifestation is very common in stable angina patients, we did not analyze it in our study. It is because the ST-T manifestation is relative complex. Despite of the segment duration, it always involves the amplitude and direction of the ST-T wave. The ST-T manifestation in angina patients always affected by the timing of myocardial ischemia, the myocardium area supplied by the diseased coronary artery and also due the alternation in ventricular repolarization and cardiomyocyte electrophysiology [69, 70]. Forth, if the study population had different severity and phases of heart diseases (such as stable angina versus acute coronary syndrome or chronic ischemic heart diseases), differences in clinical scenery may have different impact on the results. To avoid selection bias, we had chosen individuals with stable angina in this study, and thus our results may not be generalizable to other populations. Fifth, we did not evaluate the genetic factor in our study, possible confounding effects cannot be excluded. Finally, the underlying mechanism how FGF21 is associated with cardiomyocyte ion channel expression and electrophysiology remains unclear, further investigations are warranted to elucidate these issues.

## Conclusion

This study indicates that plasma FGF21 is associated with a wide QRS complex and prolonged corrected QT interval in patients with stable angina. However, the underlying mechanisms by which FGF21 result in prolonged QRS duration and QTc interval remain unclear and need to be investigated.

## Supplementary Information

**Additional file 1:** Laboratory measurements, Angiographic definitions, Electrocardiography, QT and QTc interval measurements.

## Data Availability

The datasets used and/or analysed during the current study are available from the corresponding author on reasonable request.

## Abbreviations

CAD: Coronary artery disease
LDL-C: Low-density lipoprotein cholesterol
BBB: Bundle branch block
QTc: Corrected QT
FGFs: Fibroblast growth factors
FGFR: FGF receptor
Sirt1: Silent information regulator 1
MI: Myocardial infarction
MACEs: Major adverse cardiovascular events
BMI: Body mass index
TGs: Triglycerides
HDL-C: High-density lipoprotein cholesterol
HbA1c: Hemoglobin A1c
ALT: Alanine aminotransferase
AST: Aspartate aminotransferase
ELISA: Enzyme-linked immunosorbent assay
hs-CRP: High sensitivity C-reactive protein
SBP: Systolic blood pressure
DBP: Diastolic blood pressure
LVH: Left ventricular hypertrophy
eGFR: Estimated glomerular filtration rate
CKD-EPI: Chronic kidney disease epidemiology collaboration
SD: Standard deviation
IQR: Interquartile range
ORs: Odds ratios
CI: Confidence interval
BUN: Blood urine nitrogen
T2DM: Type 2 diabetes mellitus
NAFLD: Nonalcoholic fatty liver disease
LVEF: Left ventricular ejection fraction
