# Supplementary material for: Fibroblast growth factor 21 is associated with widening QRS complex and prolonged corrected QT interval in patients with stable angina
Source: BMC Cardiovasc Disord. 2022 Sep 30;22:432. doi: 10.1186/s12872-022-02868-3 (PMC9523937; doi:10.1186/s12872-022-02868-3)
Supplement: Supplementary file 1 — Additional file 1: Laboratory measurements, Angiographic definitions, Electrocardiography, QT and QTc interval measurements. [file 12872_2022_2868_MOESM1_ESM.doc]

**SUPPLEMENTARY DATA**

**Fibroblast growth factor 21 is associated with widening QRS complex and prolonged corrected QT interval in patients with stable angina**

**Contents**

Laboratory measurements……………………………………………………………………2

Angiographic definitions………………………………………………………………………3

Electrocardiography, QT and QTc interval measurements………………………………..4

**Laboratory measurements**

Venous blood samples were drawn for biochemical analysis after a fasting period of at least 8 h. All biochemical analyses were carried out within 2 h of blood sampling at the E-Da Hospital laboratory. Serum creatinine was analysed by means of the Kinetic Jaffé’s method on a SYNCHRON CX System analyser (SYNCHRON, Los Angeles, CA) using reagents from Beckman (Beckman Coulter Diagnostic, Los Angeles, CA). Serum triglycerides, total cholesterol, low-density lipoprotein (LDL) cholesterol, high-density lipoprotein (HDL) cholesterol, uric acid, albumin, serum urea nitrogen and glucose were determined by standard commercial methods on a parallel- multichannel analyser (SYNCHRON, Los Angeles, CA) as our previous reports [26]. The inter-assay coefficients of variation were 2.1% for creatinine, 4.1% for triglycerides, 2.2% for total cholesterol, 5.5% for LDL cholesterol, 4.9% for HDL cholesterol, 1.7% for glucose, 3.1% for blood urea nitrogen and 3.2% for albumin. The blood biochemistry analyses were under internal and external quality control at the laboratory according to the College of American Pathologists’ surveys. Furthermore, the plasma level of high molecular weight adiponectin was determined using a commercial solid phase ELISA kit (B-Bridge International, Sunnyvale, CA). The dilution curve was parallel to the standard curve. The inter- and intra-assay coefficients of variation of the assay were 3.2-7.3% (n = 3) and 3.1-6.2% (n = 4), respectively. A high-sensitivity method was used to measure levels of plasma CRP with an IMMAGE system (Beckman Coulter, Immunochemistry Systems, Brea, CA, USA) that had a detection limit of 0.2 mg/L. The intra-assay coefficient of variation was 4.2% to 8.7% for hs-CRP. Samples were assessed in duplicate in a single experiment.

**Angiographic definitions**

Angiograms and quantitative coronary angiographic (QCA) analyses were scored according to one of the scoring systems and Gensini scoring: (1) in each case, coronary angiography was performed in standard projections for different coronary arteries (Philips Integris Allura 9/9 bi-plane systems). QCA analysis was performed by at least 2 experienced interventional cardiologists blinded to clinical information and serologic parameters. CAD with ≥75% stenosis in 3 main coronary arteries supplying the myocardium was confirmed by coronary angiography and was classified as having single-, 2- or 3-vessel disease. If the diameter of stenosis of the left main coronary artery exceeded 50%, it was excluded due to an undefined number of diseased vessels; (2) In the modified Gensini scoring system, weights are assigned to each coronary segment depending on the vessel size and importance, ranging from 0.5 to 5.0; segments serving larger regions of the myocardium are more heavily weighted. The narrowing of the coronary artery lumen is scored 2 for 0% to 25% stenosis, 4 for 26% to 50%, 8 for 51% to 75%, 16 for 76% to 90%, 32 for 91% to 99%, and 64 for 100%. The modified Gensini index is the sum of the total weights for each segment [31].

**Electrocardiography, QT and QTc interval measurements**

Briefly, standard resting 12-lead body-surface ECG was recorded for each patient with a paper speed of 25 mm/s and the gain set at 10 mm/mV to ensure a clear, stable baseline with no interference. All ECG patterns were evaluated according to commonly adopted clinical criteria (references1-3) . Heart rate was defined as average heart beat per minute. The PR interval is measured from the beginning at the T-P junction at the start of P wave to the initiation of the QRS segment. A Q wave is any downward deflection after the P wave. An R wave follows as an upward deflection, and the S wave is any downward deflection after the R wave. The QRS duration was then is the time interval from the onset to the end of the QRS complex. QT interval is defined as the interval between the first deflection of the QRS complex and the end of the T wave. The detail measurement of QTc was explained below. All the R, S and T wave amplitudes of the 12 leads in ECG was measured from the isoelectric line (PR segment level) to the related peak of the waves [32-34].

The QTc intervals were calculated by the Bazett's formula (QTc=QT/√RR). The ECG tracing were blinded to participant demography and analyzed by two independent coder. Inter reader discrepancies were resolved by direct comparison and adjudication by another supervisor. Interreader reproducibility assessment for the QT measurement found a coefficient of reliability of 0.995 and Pearson's correlation coefficient 0.995. Comparing interreader QT measurement by paired *t*-test did not achieve statistical significant (p=0.35 ). Extremely rapid (>150 bpm) and extremely slow (<40 bpm) heart rate recordings were excluded to eliminate the influence of heart rate on QT measurements [35,36].
